# Progress and perspectives of enzymatic preparation of human milk fat substitutes

**DOI:** 10.1186/s13068-022-02217-8

**Published:** 2022-11-04

**Authors:** Zeqing Liu, Lingmei Dai, Dehua Liu, Wei Du

**Affiliations:** 1 Key Laboratory for Industrial Biocatalysis, Ministry of Education, Department of Chemical Engineering, Tsinghua University, Beijing, 100084 China; 2 Tsinghua Innovation Center in Dongguan, Dongguan, 523808 Guangdong China

**Keywords:** Lipase, Human milk fat, Human milk fat substitute, Infant formula, Specificity

## Abstract

Human milk fat substitutes (HMFS) with triacylglycerol profiles highly similar to those of human milk fat (HMF) play a crucial role in ensuring the supply in infant nutrition. The synthesis of HMFS as the source of lipids in infant formula has been drawing increasing interest in recent years, since the rate of breastfeeding is getting lower. Due to the mild reaction conditions and the exceptionally high selectivity of enzymes, lipase-mediated HMFS preparation is preferred over chemical catalysis especially for the production of lipids with desired nutritional and functional properties. In this article, recent researches regarding enzymatic production of HMFS are reviewed and specific attention is paid to different enzymatic synthetic route, such as one-step strategy, two-step catalysis and multi-step processes. The key factors influencing enzymatic preparation of HMFS including the specificities of lipase, acyl migration as well as solvent and water activity are presented. This review also highlights the challenges and opportunities for further development of HMFS through enzyme-mediated acylation reactions.

## Introduction

HMFS is one kind of structured lipids designed to resemble triacylglycerols (TAGs) in HMF in terms of fatty acid composition and distribution. It not only provides 40–55% of the dietary energy and essential fatty acids, but also helps the absorption of other nutrients, the development of brain and so on [1, 2]. However, the compositions of triacylglycerols in vegetable oils differ greatly from those contained in HMF. High palmitic acid (PA, C16:0) content at sn-1,3 positions will lead to the formation of calcium soap, further cause loss of minerals, constipation, abdominal pain and intestinal blockage [3, 4]. Therefore, the preparation of HMFS with high similarity to HMF has received extensive attention.

It is recognized that HMF contains more than two hundred kinds of fatty acids, in which palmitic acid (20–30%), oleic acid (OA, C18:1) (25–40%) and linoleic acid (LA, C18:2) (10–30%) are the most abundant [2, 4–6]. About 60–70% palmitic acid is esterified at sn-2 position, whereas sn-1,3 positions are mainly occupied by unsaturated fatty acids. This profile corresponds to the digestive characteristics of infants, thus can ensures proper absorption of lipids and other nutrients. Besides these long chain fatty acids (LCFAs), medium chain fatty acids (MCFAs) and long chain polyunsaturated fatty acids (PUFAs) also have a positive impact on infant development, although their contents are usually lower than 1% [7, 8]. Medium chain fatty acids tend to be distributed on the glycerol backbone together with long chain fatty acids, mainly in the form of medium- and long-chain triglycerides (MLCTs) [9, 10]. It should be noted that both the composition and distribution of fatty acids have a valuable function for infants. Mimicking such complex compositions and unique structures is a major challenge in HMFS synthesis.

Due to the mild reaction conditions and high selectivity of enzymes, lipase-mediated preparation of HMFS is preferred and has been drawing increasing interest in recent years [4, 11]. The selectivity of lipase makes it possible to synthesize HMFS with desirable fatty acid composition and distribution. Apart from enzyme itself, different enzymatic synthetic route as well as the reaction medium and acyl migration have been demonstrated to play a significant role in the preparation of HMFS. In this review, the related progress and the perspective of lipase-mediated production of HMFS are presented.

## Different enzymatic approaches for HMFS production

Enzymatic production of HMFS can be classified as one-step, two-step and multiple-step process, and different approach has its own merits and challenges as elaborated below.

### One-step acidolysis/transesterification process

**Fig. 1:**
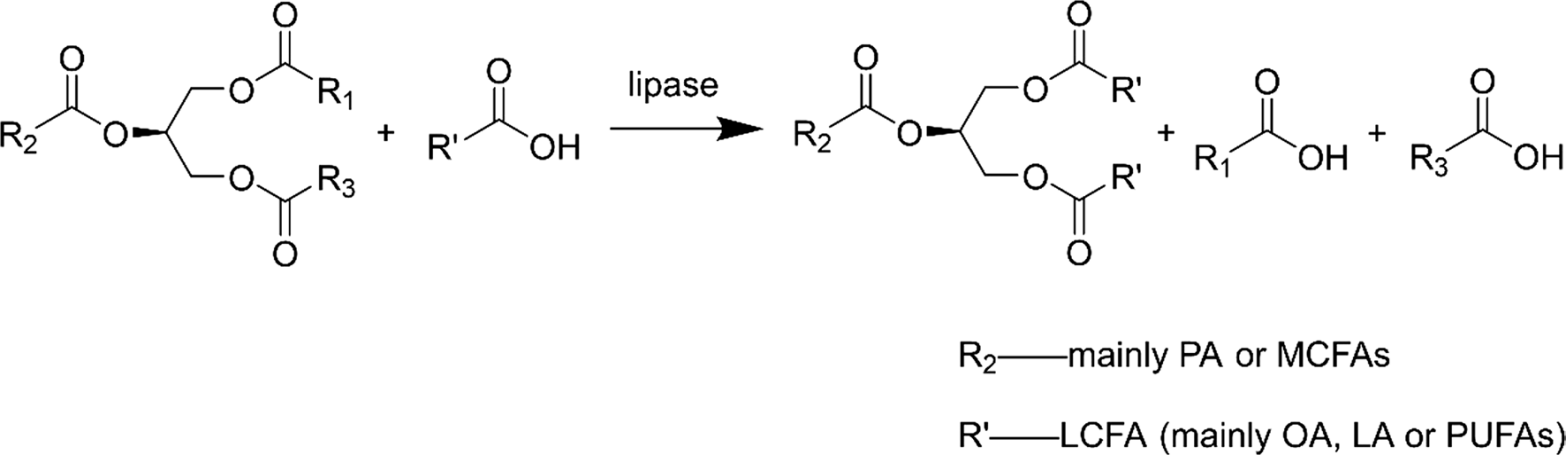
Enzymatic acidolysis for the synthesis of HMFS

Enzymatic one-step process for HMFS synthesis mainly comprises one-step acidolysis or one-step transesterification [3, 4, 12]. The acidolysis reaction is generally carried out between triacylglycerols (tripalmitin (PPP) or oil/fat rich in palmitic acid/medium chain fatty acids at sn-2 position) with free fatty acids (FFAs) (generally LCFAs, such as oleic acid, linoleic acid and polyunsaturated fatty acids), and the typical reaction is shown in Fig. 1. This one-step acidolysis reaction has been widely used for the synthesis of various types of HMFS and in terms of the types of HMFS, one-step acidolysis has been adopted for the production of 1-oleoyl-2-palmitoyl-3-oleoylglycerol (OPO), 1-oleoyl-2-palmitoyl-3-linoleoylglycerol (OPL), HMFS contained polyunsaturated fatty acids, and MLCT-type HMFS, as summarized in Table 1.

**Table 1:** HMFS synthesis through one-step acidolysis

Product type	Substrates		Enzyme (dosage)	Yield	Ref.
OPO/OPL	Fractionated palm stearin	OA + LA	NS 40,086 (8%)	sn-OPO, sn-OPL yield: 32.48%, 25.49%; sn-2 PA content: 87.75%	[15]
	Fractionated palm stearin	OA + LA	NS 40,086 (10%)	OPL content: 45.8%	[3]
				OPL content: 43.7%	
	Tripalmitin	FFAs from tea camellia seed oil	Lipozyme RM IM (10%)	sn-2 PA content: 94.51%, sn-1,3 OA content: 69.01%	[26]
	Fractionated palm stearin	OA + LA	Lipozyme RM IM (8%)	OPL content: 48.37%; sn-2 PA content: 84.70%	[27]
HMFS contained PUFAs	Tripalmitin	FFAs from camelina oil	Heterologous *Rhizopus oryzae* lipase immobilized on Lewatit VP OC 1600 (8%)	HMFS yield: 52%	[16]
	Tripalmitin	FFAs from microalgal oil	Lipozyme RM IM (90 mg)	sn-2 PA content: 89.0%, sn-1,3 PUFA content: 81.3%	[28]
	TAGs from *Nannochloropsis oculata*	FFAs from *Isochrysis galbana*	Novozym 435/Lipozyme TL IM/Lipozyme 435/Lipozyme RM IM (10%)	PUFA content: 13.92–17.12%; 59.38–68.13% PA at sn-2 position	[18]
MLCT-type HMFS	*Cinnamomum camphora* seed oil	OA	Lipozyme RM IM (12.5%)	> 70% of MCFAs at sn-2 position, 78.69% OA at sn-1,3 positions	[29]
	*Cinnamomum camphora* seed oil	OA	Lipozyme RM IM (10%)	OMO yield: 64.45%	[9]
	Algal oil	lauric acid	Lipozyme AOAB8 (12%)	Lauric acid, DHA content: 30.91%, 44.68%	[24]

OPO and OPL are the 2 most abundant triacylglycerols in HMF [2, 5]. Numerous studies have been done on the synthesis of OPO but few studies related to OPL preparation, despite of the fact that it has been found that the content of OPL is even higher than that of OPO in some special regions, such as China [3, 13, 14]. Wang et al. [15] explored the possibility to prepare structured lipids rich in OPO and OPL by lipase-catalyzed acidolysis of fractionated palm stearin with oleic acid and linoleic acid.

Apart from OPO and OPL, polyunsaturated fatty acids, such as *α*-linolenic acid (ALA, C18:3), arachidonic acid (AA/ARA, C20:4), eicosapentaenoic acid (EPA, C20:5) and docosahexaenoic acid (DHA, C22:6), also play a crucial role for the structural, functional, neurological, and cognitive development of children [16, 17]. Herein, HMFS enriched with polyunsaturated fatty acids is of great interest as infant formulas. Fish oils [13], microbial oils [18], fungal oils [19] and plant oils [16] are the common sources for polyunsaturated fatty acids supplement. However, unsaturated fatty acids especially polyunsaturated fatty acids are prone to oxidation at high temperature, thus the reaction temperature and the stability of the products need to be taken into account [20]. Abed et al. [21] synthesized 1,3-dioleoyl-2-arachidonoylglycerol-rich structured lipids from microbial oil and oleic acid via Lipozyme RM IM-catalyzed acidolysis. Faustinl et al. [16] prepared HMFS rich in polyunsaturated fatty acids by acidolysis between tripalmitin and free fatty acids from camelina oil.

Medium chain fatty acids are also essential for infants, which help to induce the residual glyceride lipolysis and thus improve fat absorption [10, 22–24]. MLCT-type HMFS has become an attractive product, since it combines the benefits of medium chain fatty acids and long chain fatty acids. Peng et al. [9] synthesized OMO (1,3-oleic-2-medium chain)-type HMFS through Lipozyme RM IM-catalyzed acidolysis between *Cinnamomum camphora* seed oil and oleic acid. Zou et al. [25] produced MLCTs with high levels of lauric acid (C12:0) and DHA via an acidolysis reaction between algal oil and lauric acid.

**Fig. 2:**
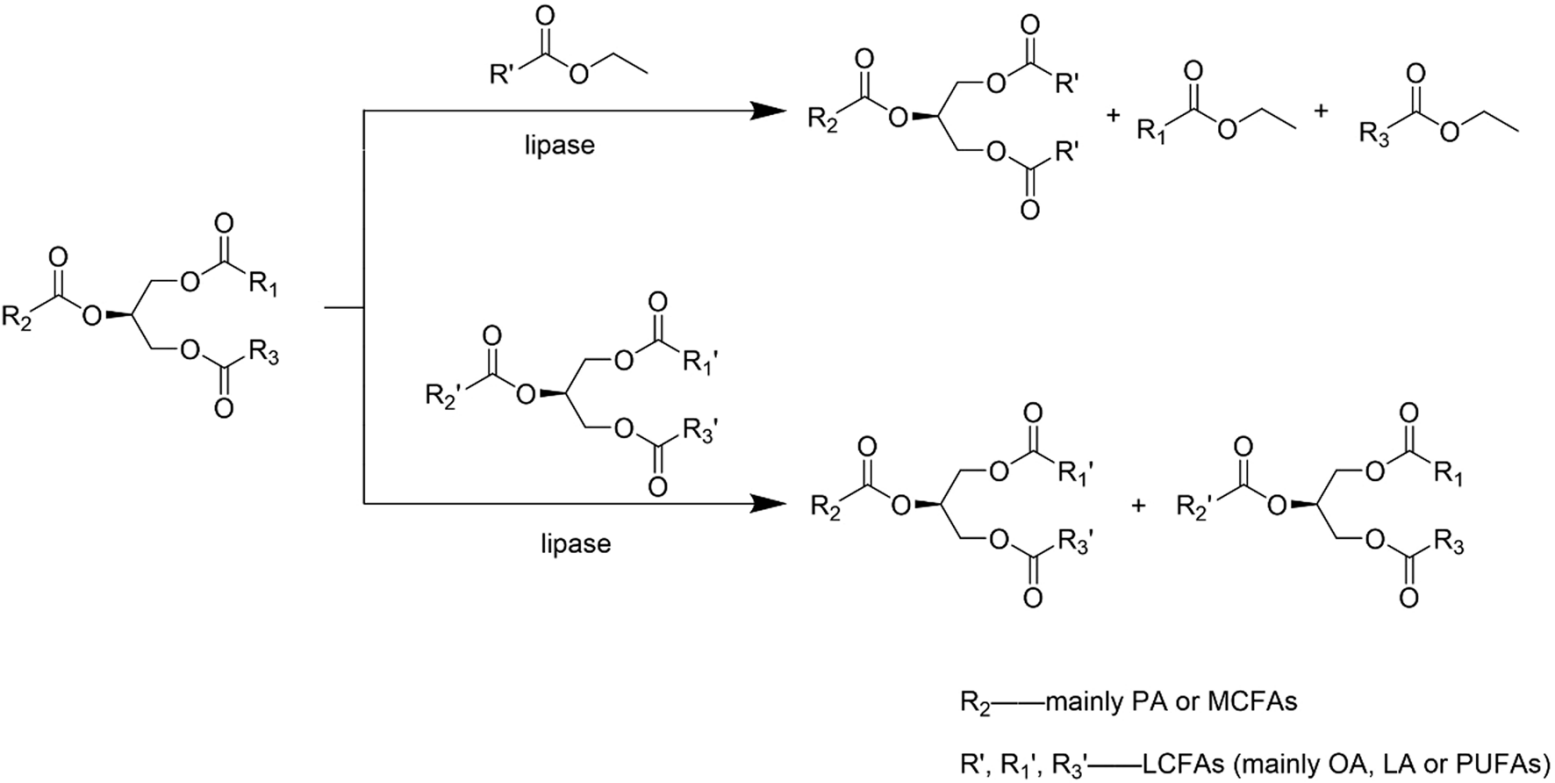
Enzymatic transesterification for the synthesis of HMFS

In addition to one-step acidolysis, one-step transesterification is an alternative for HMFS synthesis with the typical reaction shown in Fig. 2. This process is normally performed between triacylglycerols and fatty acid esters or between different triacylglycerols. OPO, MLCT-type HMFS and other HMFS similar to HMF have been prepared successfully through this one-step transesterification process and the related research is summarized in Table 2.

**Table 2:** HMFS synthesis through one-step transesterification

Type of acyl donor	Substrates		Enzyme (dosage)	Yield	Ref.
Fatty acid ester	Tripalmitin	Ethyl oleate	Immobilized *Candida parapsilosis* Lipase/Acyltransferase (5%)	OA incorporation: 51 mol%; sn-2 OA content: 15%	[38]
	Palm stearin oil	Ethyl oleate	Lipozyme RM IM (10%)	OPO content: 29.1%; sn-2 PA content: 54.5%, sn-1,3 OA content: 73.8%	[39]
	Palm stearin	Peanut oil fatty acid ethyl esters	Novozym 40,086 (8%)	OPO content: 46.30%; 60.70% PA at sn-2 position	[40]
	Tibet yak ghee	Sunflower oil ethyl esters and ethyl oleate	Lipozyme RM IM (10%)	LA content: 17.59%; 56.40% PA at sn-2 position	[30]
Oil/fat	Medium-chain triacylglycerols	ARA-rich fungal oil	Lipozyme 435 (8%)	MLCTs content: 53.75%	[10]
	TAGs rich in OPO and OPL	Coconut oil	NS 40,086 (8%)	MLCTs content: 70.44%	[22]
	Catfish oil	Coconut oil	NS 40,086 (8%)	MLCTs content: 62.14%; relative content of PA at sn-2 position: 46.14%	[23]
	Lard	Sunflower oil + canola oil + palm kernel oil + palm oil + algal oil + microbial oil	Lipozyme RM IM (11%)	Similarity in total and sn-2 fatty acid, PUFA, TAG composition: 92.5, 90.3, 61.5, 71.9	[41]
	Lard	Sunflower oil + canola oil + palm kernel oil + palm oil + algal oil + microbial oil	Lipozyme RM IM (25 g)	Degree of similarity in TAG composition: 72.3; 39.2% PA at sn-2 position; ARA, DHA content: 0.5%, 0.3%	[33]

During the transesterification process of triacylglycerols with fatty acid esters for HMFS preparation, fatty acid ethyl esters (FAEEs) is usually adopted as the acyl donor. Chen et al. [30] compared two types of acyl donors, namely, fatty acid and fatty acid ethyl ester, and they found that the degree of acyl migration was lower with fatty acid ethyl ester. A similar phenomenon was also observed by other researchers [31, 32]. Transesterification between different natural oils/fats is mainly used for the synthesis of MLCT-type HMFS and HMFS similar to HMF. Yuan et al. [23] obtained structured lipids enriched with medium chain fatty acids and high content of palmitic acid at sn-2 position via one-step transesterification, where catfish oil and coconut oil were used as the sources of palmitic acid and medium chain fatty acids. Zou et al. [33] prepared HMFS having high similarity to HMF by Lipozyme RM IM-catalyzed one-step transesterification between lard with different oils.

The composition of acidolysis products is easy to predict [1, 34], but the unavoidable acyl migration will cause undesirable fatty acid distribution [35, 36]. For one-step transesterification process, fatty acid ester as the acyl donor has been proved to be an effective strategy to increase the conversion and suppress acyl migration [30, 32, 37]. In terms of the substrate sources, natural oils/fats as the acyl donors have significant economic advantages, but it is difficult to recover the fraction of triacylglycerols resembling HMF from the reaction mixture.

### Two-step methods

Despite the simplicity of one-step methods, there are still obvious challenges limiting their industrialization, such as low yield, high requirement for raw materials (e.g., need high content of sn-2 palmitic acid) [37, 42]. Researchers attempted to achieve higher yield and expand the available range of substrates by two-step approaches, mainly acidolysis–acidolysis, acidolysis–transesterification, alcoholysis–acidolysis, alcoholysis–transesterification, esterification–esterification and esterification–acidolysis processes. These methods are mainly used for the synthesis of OPO and HMFS containing polyunsaturated fatty acids (Table 3).

**Table 3:** Studies on the synthesis of HMFS by two-step method

Reaction type	Step 1		Step 2		Yield	Ref.
	Substrates	Catalyst	Substrates	Catalyst		
Acidolysis–acidolysis	Tuna oil/PA	Novozym 435	TAGs/FFAs rich in OA	Lipase DF	sn-2 PA, sn-2 DHA content: 52.1%, 15.4%, sn-1,3 OA content: 67%	[44]
Transesterification–transesterification	Hazelnut oil + ethyl palmitate	Novozym 435	TAGs/ARA/DHA-rich single-cell oils	Lipozyme RM IM	PA, ARA, DHA content: 58.9%, 2.7%, 1.5%; sn-2 PA content: 62.5%	[43]
Acidolysis–transesterification	Camellia seed oil + PA	Novozym 435	TAGs/fish oil	Lipozyme TL IM	PA, EPA, DHA, ARA content: 27.88%, 14.50%, 7.38%, 0.99%; sn-2 PA content: 55.97%	[49]
Alcoholysis–transesterification	Tripalmitin + ethanol	*Candida* sp. 99–125 lipase	2-MAG/ethyl oleate	*Candida* sp. 99–125 lipase	OPO content: 85.06%; sn-2 PA content: 80.17%	[46]
Alcoholysis–acidolysis	Lard + ethanol	Novozym 435	(Lard + 2-MAG)/(OA + LA)	Lipozyme RM IM	TAG recovery: 66.2 mol%; sn-2 PA content: 66.8%	[45]
	Fish oil + ethanol	Novozym 435	2-MAG/OA	Lipozyme RM IM	sn-1,3-OA-sn-2-PUFA-TAG yield: 86.3%	[50]
	Lard + ethanol	Novozym 435	(Lard + 2-MAG)/(OA/LA/Ln, 5:2:1, mol)	Lipozyme RM IM	Non-PUFA-containing HMFS yield: 71.3%	
Alcoholysis–(acidolysis and transesterification)	Lard + ethanol	Novozym 435	(Lard + 2-MAG)/(OA/LA/Ln, 5:2:1, mol, and PUFA-EEs)	Lipozyme RM IM	sn-1,3-PUFA-HMFS (10% PUFAs at sn-1,3 positions) yield: 69.4%	
Esterification–esterification	Glycerol + OA	Novozym 435	Products in step 1/PA	Novozym 435	OPO yield: 15.02%; C52 content: 38.55 wt%; sn-2 PA content: 39.06%	[47]
Esterification–acidolysis	Glycerol + PA	NaOH	Tripalmitin/OA	Lipozyme RM IM	OPO content: 40.23%; 86.62% PA at sn-2 position, 63.74% OA at sn-1,3 positions	[48]
				Lipozyme TL IM	OPO content: 32.34%; 92.92% PA at sn-2 position	

**Fig. 3:**
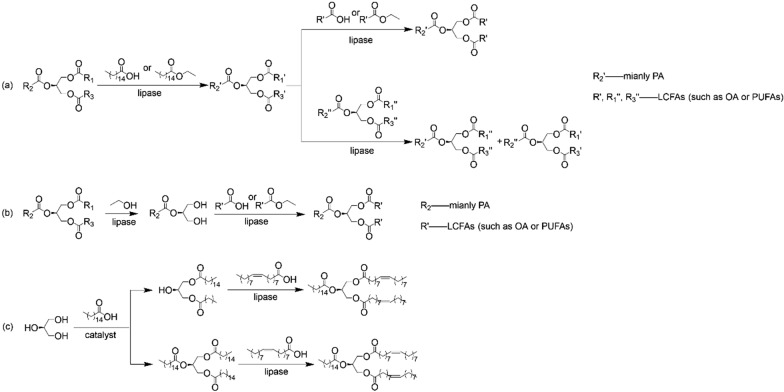
Two-step processes for the synthesis of HMFS

Acidolysis–acidolysis and acidolysis–transesterification are the most common strategies in two-step processes for the synthesis of HMFS, Fig. 3a. Turan et al. [43] prepared palmitic acid-enriched hazelnut oil via Novozym 435-catalyzed transesterification of hazelnut oil and ethyl palmitate. Then, HMFS containing a high amount of palmitic acid at sn-2 position and long-chain polyunsaturated fatty acids (DHA and ARA) at sn-1,3 positions was produced through the second-step Lipozyme RM IM-catalyzed transesterification. Roble et al. [44] carried out a two-step acidolysis for the synthesis of structured lipids rich in palmitic acid and DHA at sn-2 position and oleic acid at sn-1,3 positions. First, triacylglycerols with high content of palmitic acid and DHA were obtained via acidolysis of tuna oil and palmitic acid, catalyzed by a non-positional specific lipase Novozym 435. Then, fatty acids located at sn-1,3 positions were replaced through the second-step acidolysis catalyzed by a sn-1,3-specific lipase DF.

Alcoholysis–acidolysis and alcoholysis–transesterification have also been explored for the synthesis of HMFS, Fig. 3b. At first, alcoholysis of triacylglycerols substrate catalyzed by a sn-1,3 specific lipase was conducted to produce 2-monoglyceride (MAG) rich in palmitic acid. Then, HMFS rich in palmitic acid at sn-2 position was synthesized through the second-step acidolysis or transesterification with fatty acids or fatty acid esters. HMFS containing 66.8% palmitic acid at sn-2 position was prepared through Novozym 435-catalyzed ethanolysis of lard in the first step and acidolysis for the second-step [45]. OPO with 80.17% palmitic acid located at sn-2 position was synthesized through alcoholysis–transesterification process [46]. In this process, 2-MAG was prepared though the alcoholysis of tripalmitin, followed by molecular distillation separation and then 2-MAG with high purity was further used for OPO preparation through the second-step transesterification reaction. Esterification–esterification and esterification–acidolysis are other alternatives for the production of HMFS, Fig. 3c. Agapay et al. [47] explored the esterification–esterification process for OPO production using Novozym 435 as the biocatalyst. Although the regioselectivity can be manipulated by varying reactant feeding scheme, the use of non-specific lipase resulted in relatively low OPO yield. Wei et al. [48] proposed a facial esterification–acidolysis process to prepare structural triglycerides rich in PA at sn-2 position. First, PPP was obtained by esterification of PA and glycerol with NaOH as catalyst, then OA was incorporated into the PPP glycerol backbone by acidolysis catalyzed using sn-1,3 regiospecific lipases.

### Multi-step method

**Fig. 4:**
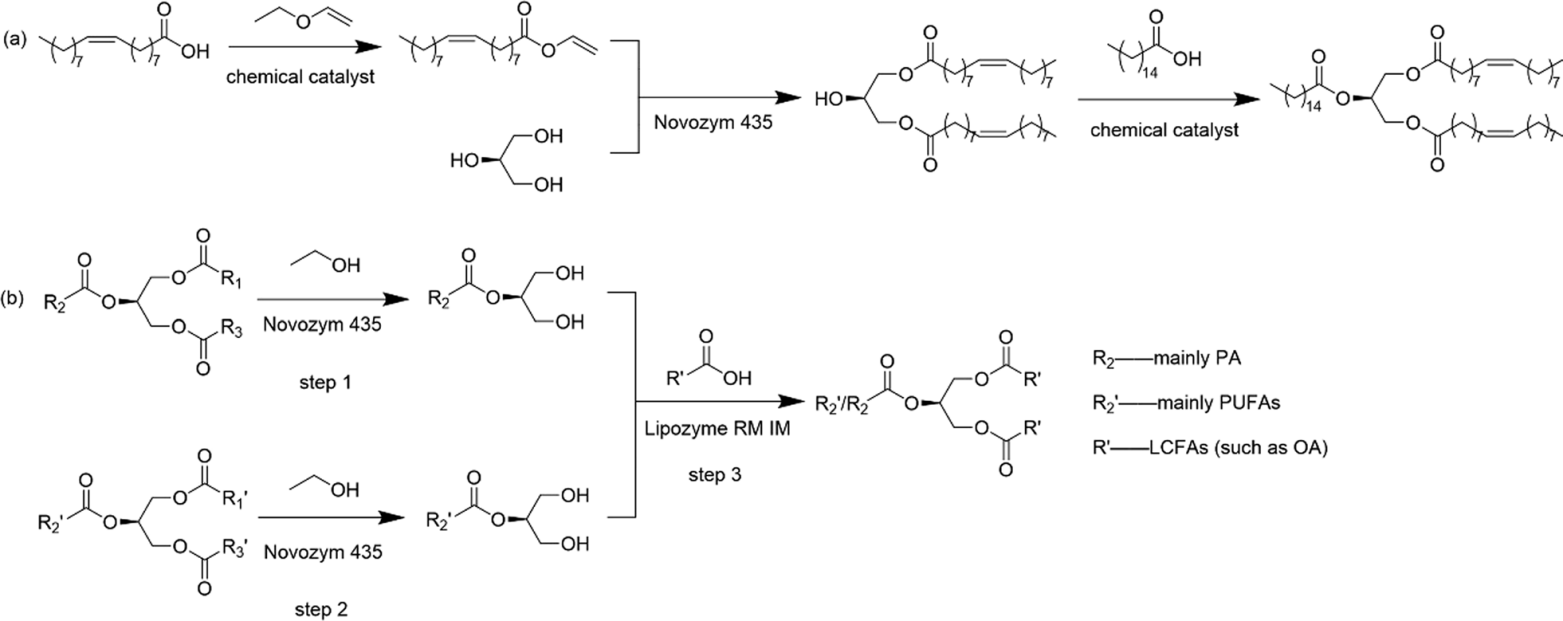
Multi-step process for the synthesis of HMFS

Regarding multi-step processes for HMFS production, few studies have been carried out. Wang et al. [51] prepared sn-OPO with high purity by a three-step method, Fig. 4a, with 90.5% yield and 98.7% regiopurity obtained. First, vinyl oleate was chemically prepared by transvinylation between vinyl acetate and oleic acid. Then, vinyl oleate further reacted with glycerol to produce 1,3-diolein with Novozym 435 as the biocatalyst. Finally, sn-OPO was chemically synthesized by esterification between 1,3-diolein and palmitic acid. The irreversible acylation route for the synthesis of 1,3-diolein and OPO were considered as the main novelties of this study. Ogasawara et al. [50] synthesized HMFS containing polyunsaturated fatty acids selectively at sn-2 position by a three-step process, Fig. 4b. At first, 2-PA-MAG and 2-PUFA-MAG were prepared by Novozym 435-catalyzed ethanolysis using lard and fish oil as the substrates (step 1 and 2). Then, sn-2-PUFA-HMFS was synthesized by Lipozyme RM IM-catalyzed reaction, using lard, 2-PA-MAG, 2-PUFA-MAG and long chain fatty acids as mixed substrates (step 3). The physiological performance and oxidative stability indicated that product with polyunsaturated fatty acids mainly distributed at sn-2 position was more desirable.

Different approaches have their own advantages and challenges. Generally speaking, compared to one-step process, two-step approaches for HMFS preparation can broaden the range of available substrates and improve the purity of HMFS product, but the complexity of the steps potentially leading to an increased overall cost needs to be taken into consideration. For multi-step methods, the complexity of multiple steps and large amount of lipase required limit their industrial application to a great extent. Apart from different enzymatic approach influencing the production of HMFS, some factors, such as lipase species, reaction medium and acyl migration also play a significant role in the enzymatic acylation processes.

## Key factors influencing enzymatic HMFS production

### Substrate specificity of lipase

The substrate specificities of lipase in catalyzing natural triacylglycerols’ acylation reaction can be mainly classified as regioselectivity and fatty acid selectivity, which have been demonstrated to play a decisive role in enzymatic synthesis of HMFS [52]. The lipase’s regioselectivity and fatty acid selectivity may be attributed to the differences in structure, size and chemical properties [53]. The regioselectivity of lipase determines the distribution of fatty acids in HMFS. Triacylglycerols of unsaturated–saturated-unsaturated (USU) type are the majority in HMF and many lipases with regioselectivity have been investigated as the catalysts for HMFS production [54, 55] (Table 4).

**Table 4:** Regioselectivity of some lipases

Source organism	Regioselectivity
*Aspergillus niger*	sn-1,3 Regioselectivity
*Aspergillus oryzae*	sn-1,3 Regioselectivity
*Rhizopus oryzae*	sn-1,3 Regioselectivity
*Rhizopus niveus*	sn-1,3 Regioselectivity
*Rhizomucor meihei*	sn-1,3 Regioselectivity
*Candida* sp. 99–125	sn-1,3 Regioselectivity
*Candida parapsilosis*	sn-1,3 Regioselectivity
*Thermomyces lanuginosa*	sn-1,3 Regioselectivity
*Yarrowia lipolytica*	sn-1,3 Regioselectivity
*Bacillus thermocatenulatus*	sn-1,3 Regioselectivity
Porcine pancreatic	sn-1,3 Regioselectivity
*Candida antarctica lipase B*	Non-regioselectivity
*Candida rugosa*	Non-regioselectivity
*Burkholderia cepacia*	Non-regioselectivity

Lipases with sn-1,3 regioselectivity are necessary for the synthesis of HMFS, such as Lipozyme RM IM (from *Rhizomucor miehei*) [9, 20, 21], NS 40086 (from *Aspergillus oryzae*) [3, 22, 23] and Lipozyme TL IM (from *Thermomyces lanuginose*) [18, 39]. While lipases without regioselectivity, such as Novozym 435 (from *Candida antarctic* lipase B) [49, 56, 57] can help increase the content of palmitic acid in raw substrates and have also been adopted for HMFS preparation especially in the two-step or multi-step processes.

Apart from regioselectivity, lipase’s selectivity toward different fatty acids also affects the fatty acid composition of HMFS. This selectivity was found to be associated with the carbon chain length, double bond number, double bond position and even the reaction types [58, 59]. Considering the specific fatty acid composition of HMF, the related studies regarding the selectivity toward saturated fatty acids (SFAs), C18 unsaturated fatty acids and long-chain polyunsaturated fatty acids are specially reviewed and summarized in Table 5. Researchers investigated the fatty acid selectivity of lipase under various reaction types. During lipase-catalyzed esterification of fatty acids, many lipases expressed preferred selectivity toward fatty acids with medium or long carbon chain. Lee et al. [60] found that four lipases from *Burkholderia cepacia*, *Rhizomucor miehei*, *Candida antarctica* (type B) and *Candida rugosa* all showed preference for C8 fatty acid in esterification reaction. Kovalenkoa et al. [61] prepared biocatalyst by immobilizing a recombinant lipase from *Thermomyces lanuginosus* on mesoporous SiO_2_ and further studied its fatty acid selectivity during esterification. The immobilized lipase showed preference for fatty acids with more than seven carbon atoms. However, Novozym 435 exhibited preference for short chain fatty acids and long chain fatty acids, as reported by Wu et al. [62]. Duan et al. [63], Misbah et al. [64] and Rivero-Pino et al. [65] found that many lipases had substrate specificity toward triacylglycerols with short or medium carbon chain during the hydrolysis of triacylglycerols. While for the hydrolysis of fatty acid esters, lipases expressed different selectivity. Song et al. [59] compared the substrate specificity of nine lipases with esters as the hydrolysis substrates, and they found lipases from S_3_ *Penicillium citrinu*, MJ_1_ *Aspergillus niger*, MJ_2_ *Aspergillus oryzae*, YM *Bacillus coughing*, S_9_ *Geotrichum candidum* and S_11_ *Candida lypolytica* showed the strongest specificities to short chain esters, and the other lipases tested showed strong selectivity for medium or long chain esters. Utisugi et al. [66] and Karabulut et al. [67] found that lipases showed high selectivity for medium chain fatty acids when transesterification was conducted between triacylglycerols and various fatty acids. On the contrary, Karabulut et al. [68] studied the fatty acid selectivity of different lipases in the transesterification between oleic acid and monoacid triacylglycerols, and found that all tested lipases showed low activity for triacylglycerols with medium chain fatty acids.

**Table 5:** Studies on the substrate selectivity for fatty acids with different chain length or unsaturated degree

Difference in substrates	Reaction type/substrates	Lipase	Selectivity	Ref.
Carbon chain length	Hydrolysis/fatty acid ethyl esters (C4–C18)	Novozym 435	Short chain fatty acid ethyl esters	[62]
	Esterification/fatty acids (C4–C18) with ethanol		Short and long chain fatty acids	
	Alcoholysis/TAGs (C4–C18) with ethanol		TAGs with short chain	
	Esterification/xylose and SFAs (C12/C16)	Lipozyme 435	Non-modified L435:C16; polyethyleneimine coated L435:C12	
	Esterification/SFAs (C4–C18) with aliphatic alcohols	*Thermomyces lanuginosus* lipase immobilized on mesoporous SiO_2_	Fatty acids with more than seven carbon atoms	[69]
	Esterification/SFAs (C2–C10, C18) with aliphatic alcohols (C2–C12, C16)	*Thermomyces lanuginosus* lipase immobilized into mesoporous silica	Fatty acids with more than seven carbon atoms	[61]
	Esterification/SFAs (C7/C18) with aliphatic alcohols	r*Pichia*/lip lipase immobilized on aggregated carbon nanotubes	C7:0	[70]
	Hydrolysis/acyl p-nitrophenyl esters and TAGs (C2–C16)	*Malbranchea cinnamomea* lipase	Acyl p-nitrophenyl esters and triglycerides with short and medium carbon chain	[63]
	Hydrolysis/triglycerides (C4–C18)	Lipase from *Proteus vulgaris* OR34	Short and medium chain fatty acids	[64]
	Hydrolysis/acyl p-nitrophenyl esters (C2–C18)	Lipases from *Geobacillus thermocatenulatus*	Est29: short chain fatty acids (C4–C8); Lip29: LCFAs (C12–C16)	[71]
	Hydrolysis/esters	Lipases from different sources	Lipases from S_3_ *Penicillium citrinum*, MJ_1_ *Aspergillus niger*, MJ_2_ *Aspergillus oryzae*, YM *Bacillus coughing*, S_9_ *Geotrichum candidum*, S_11_ *Candida lypolytica*: short-chain esters; lipases from M_2_ *Mucor racemosus*, Y-11 *Trichosporon capitatum*, J_8-2_ *Rhizopus oryzae*: medium or long chain and branched esters	[59]
Double bond	Hydrolysis/cod liver and linseed oils	*Candida rugosa* lipase; *Candida antarctica* lipase *B*; *Thermomyces lanuginosa* lipase	SFA > MUFA > PUFA; all three lipases did’t discriminate against ALA	[72]
	Hydrolysis/vegetable oils	Lipase from *Penicillium citrinum* URM 4216	Unsaturated fatty acids, such as OA and LA	[73]
	Hydrolysis/high-acidity vegetable oils	Lipase from castor bean seeds	Vegetable oils rich in PUFAs	[74]
	Hydrolysis/fish oil glycerides	OUC-Lipase 6	MUFA > SFA > PUFA	[75]
	Transesterification/2-MAG with EPA-EE/DHA-EE	Novozym 435; Novozym 40,086; Lipozyme TL IM	EPA-EE	[76]

The number and position of double bonds in fatty acid influenced the lipase’s specificities in many cases. During lipase-mediated hydrolysis, the priority is usually SFAs, monounsaturated fatty acids (MUFAs) and PUFAs [75]. Lipases exhibited less catalytic activity when double bonds located nearest the carboxyl group of fatty acid, such as DHA [76]. Akanbi et al. [77] found that *Candida antarctica* lipase A showed sequential selectivity for fatty acids in the order of increasing number of double bonds during the hydrolysis of Anchovy oil. *Candida rugosa* lipase, *Candida antarctica* lipase B and *Thermomyces lanuginose* lipase were found to have the same selective regularity [72]. While some lipases were demonstrated to favor the hydrolysis of natural oils rich in polyunsaturated fatty acids [73, 74]. The selectivity of lipases for fatty acids with the same carbon chain length but different unsaturation degree has also been explored. Karabulut et al. [58] investigated the selectivity of three lipases for C18 unsaturated fatty acids in catalyzing acidolysis and found that Lipozyme TL IM, Lipozyme RM IM and Novozym 435 all had the fatty acid substrate preference in the order of C18:3 > C18:2 > C18:1. On the contrary, Corzo-Martínez et al. [78] found that immobilized lipase from *Rhizopus oryzae* showed preference for C18 with lower unsaturated degree.

**Fig. 5:**
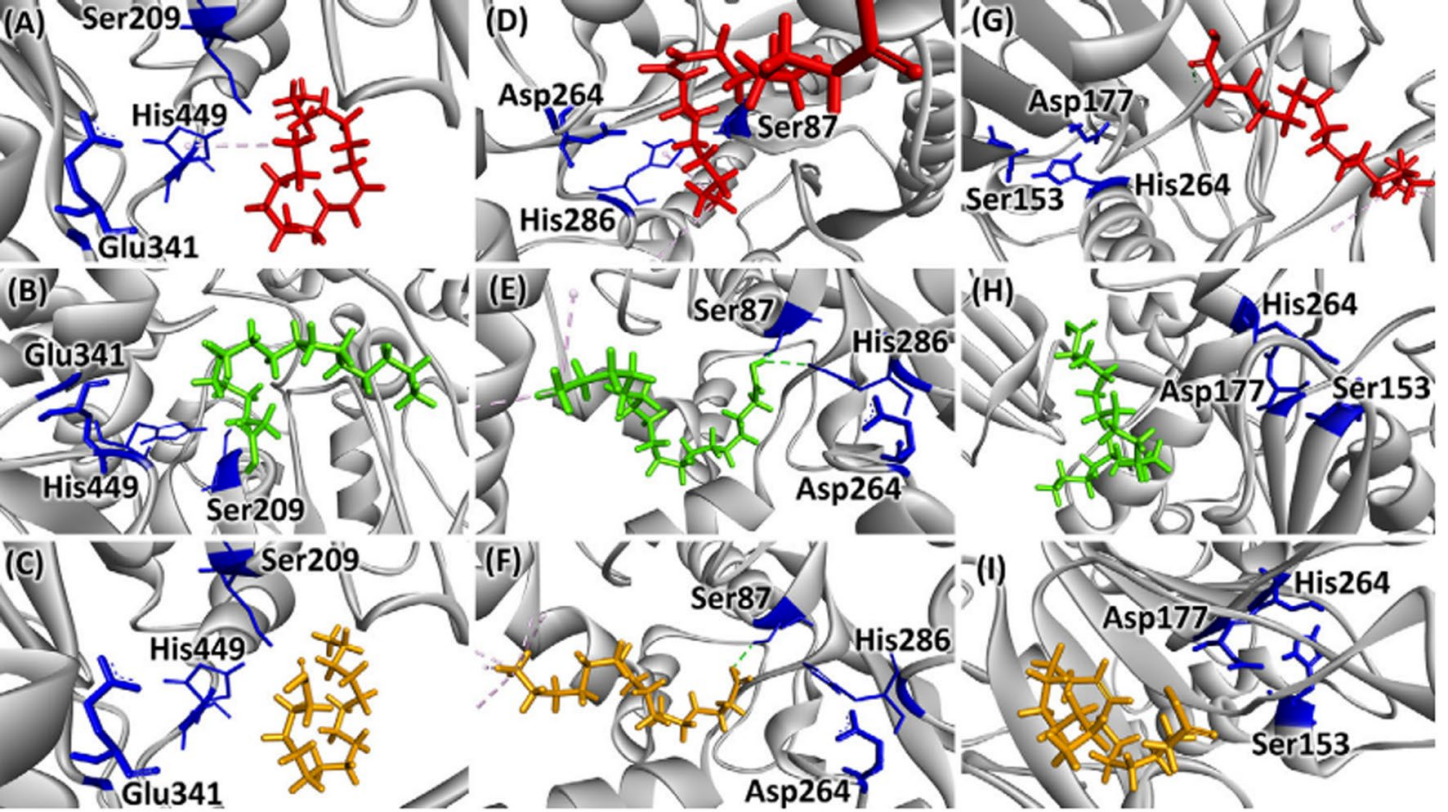
Docking pose of the fatty acids (oleic in red, stearic in green, and palmitic in orange), in the backbone peptide structure of lipases (CRL, BCL and PPL) in gray, and their catalytic triad in blue [79]

It is demonstrated well that the geometric structures and properties of enzyme and substrate determine the lipase’s substrate specificities. Thus, molecular docking can be a useful technique for binding affinity estimation, interactions analysis and substrate selectivity prediction [79–81]. Barbosa et al. [79] successfully used molecular docking analysis for the fatty acid specificity of three commercial lipases from *Candida rugosa* (CRL), *Burkholderia cepacia* (BCL), and porcine pancreas (PPL), the poses with the lowest absolute affinity value for each fatty acid docked in each lipase are displayed in Fig. 5. Three fatty acids (oleic, stearic, and palmitic acid) have negligible binding energies to *Candida rugosa* lipase, and oleic acid can interact with His449 in the *Candida rugosa* lipase active site with a higher availability, indicating the preference for long-chain unsaturated fatty acids. *Burkholderia cepacia* lipase shows a higher catalytic affinity for stearic acid, since the binding energy is lowest and stearic acid can interact directly with 2 of the 3 amino acid residues constituting the catalytic triad of *Burkholderia cepacia* lipase. However, none of the tested ligands can interact with the active site of porcine pancreas lipase, showing that its low catalytic performance. The validity of molecular simulations was also demonstrated by Rodrigues et al. [82], Cai et al. [81] and Jo et al. [71].

### Acyl migration

**Fig. 6:**
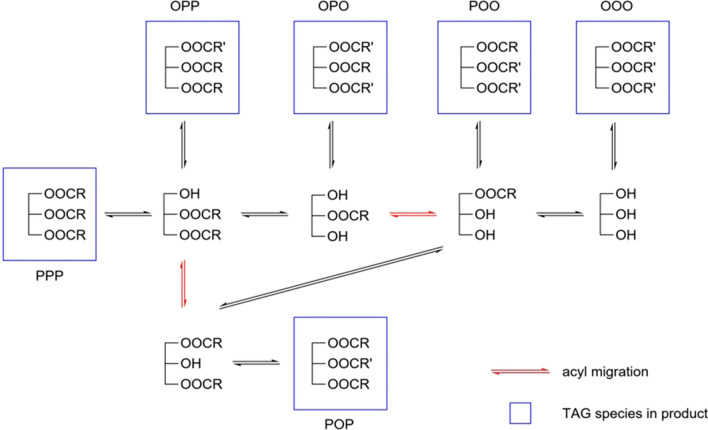
Acyl migration during lipase-mediated acidolysis between tripalmitin and oleic acid

Acyl migration is the main side reaction in enzymatic production process of HMFS, which may cause the undesirable distribution of fatty acid and cannot be completely avoided even in a highly enzymatically regiospecific reaction [83, 84]. What is more, this problem is particularly serious in lipase-catalyzed acidolysis reaction [85]. Enzymatic acidolysis is usually considered as a two-step reversible reaction, consisting of hydrolysis of ester bond and then esterification of FFA [36]. In this process, diglyceride (DAG) and MAG are the two inevitable intermediates and further act as the precursors of acyl migration. With only 2 kinds of fatty acids used for HMFS preparation, various by-products with undesirable fatty acid distribution will be formed due to the acyl migration occurring in the process, as illustrated in Fig. 6. The product composition is more complicated when more fatty acids involved for the synthesis of HMFS.

**Fig. 7:**
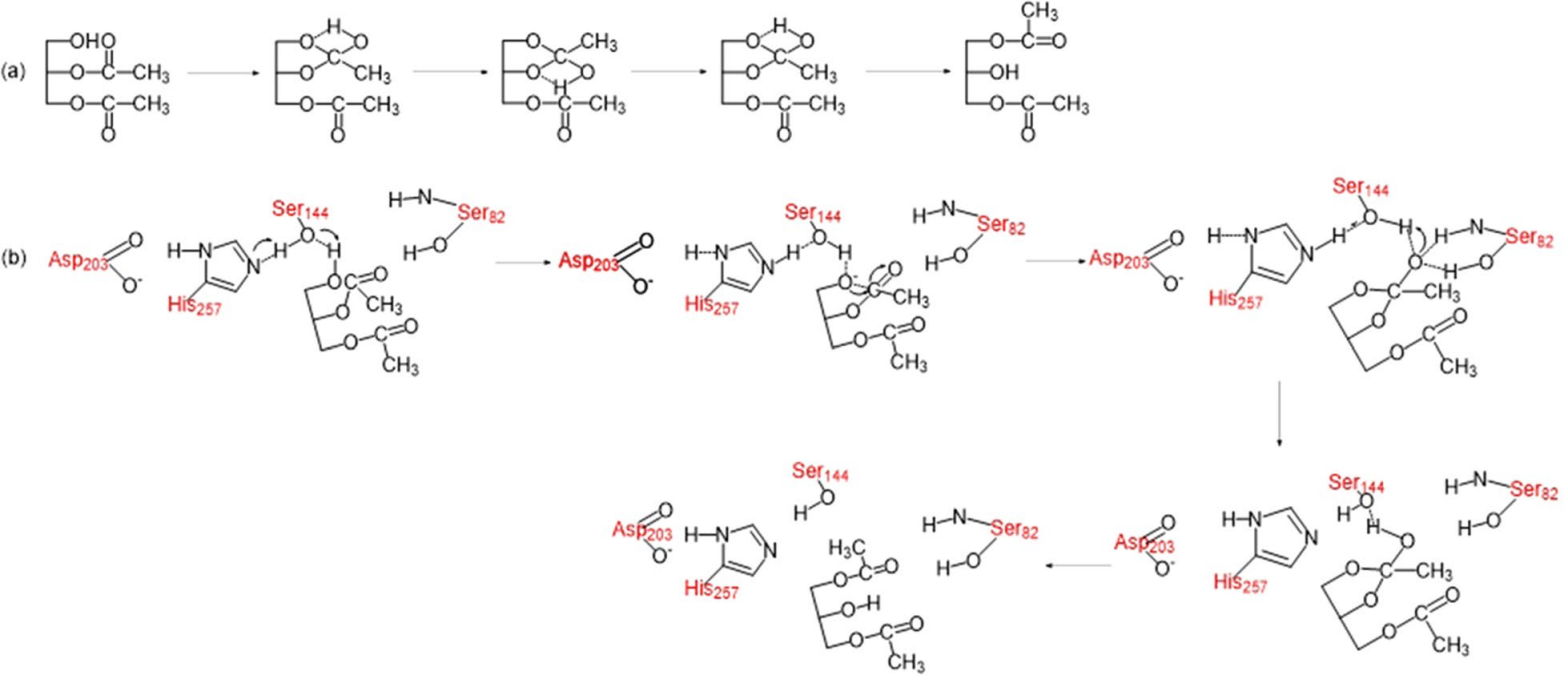
Possible mechanism of acyl migration in: **a** NCM scheme; **b** LCM scheme

The mechanisms of acyl migration can be divided into two types: (a) non-catalyzed mechanism (NCM); (b) lipase-catalyzed mechanism (LCM) [36, 84, 86]. In NCM, the postulated intramolecular acyl migration mechanism occurs via sn-2 nucleophilic substitution. After the hydrolysis of triacylglycerol, the ester carbonyl carbon will be nucleophilic attacked by electrons of the hydroxyl oxygen, resulting in an unstable five-membered cyclic intermediate, which eventually opens the ring to form the product, Fig. 7a. Some studies suggested that lipase-catalyzed acyl migration had a unique reaction pathway, and would occur simultaneously with the NCM of acyl migration [84, 87, 88]. Mao et al. [84] proposed the possible mechanism under the condition of no water molecule involved in the process, Fig. 7b. First, His257 deprotonates Ser144, while Ser144 captures the hydroxyl proton on the sn-1 position of the substrate. Then, the oxyanion on the sn-1 position will nucleophilic attack the sn-2 carbonyl carbon, resulting in a cyclic five-membered ring. Following the nucleophilic attack, the C=O double bond gradually elongates to a C–O single bond, and the newly formed oxyanion is stabilized by Ser144 and Ser82 residues. Subsequently, Ser144 is re-protonated by His257, while Ser144 protonates the newly formed oxyanion. Finally, the five-membered ring opens, accompanied by the transfer of protons from the sn-2 carbonyl carbon to the sn-1 ester oxygen. During lipase-mediated HMFS production, whether acyl migration following non-catalyzed or enzyme-catalyzed mechanism needs to be investigated especially when different lipases and different acyl donor/acceptor present in the system.

### Solvent and water activity

Solvent and water activity are also found to have profound influence on lipase-catalyzed HMFS production, which may influence the degree of acyl migration, lipase’s substrate specificity and so on. Enzymatic synthesis of HMFS can be performed in either organic solvent or solvent-free system, but due to the limitation of reactants solubility, many related researches were carried out in solvent system (Table 6).

**Table 6:** Studies on the effect of solvent on lipase performance and acyl migration

Lipase	Reaction type/substrates	Solvent	Effect	Ref.
*Candida *sp. 99–125 lipase	Esterification between glycerol and OA	Acetone; tetrahydrofuran (THF); *t*-butanol; 4-methyl-2-pentanone; chloroform; toluene; tetrachloromethane; cyclohexane; *n*-hexane; *n*-heptane; *n*-octane	Solvent with high log *P*: high activity, weak sn-1,3 selectivity	[90]
Novozym 435	Esterification between glycerol and OA	Acetone; THF; *t*-butanol; 4-methyl-2-pentanone; chloroform; toluene; tetrachloromethane; cyclohexane; *n*-hexane; *n*-heptane; *n*-octane	Solvent with high log P: high activity, weak sn-1,3 selectivity	[94]
Whole-cell lipase from *Aspergillus niger* GZUF36	Glycerolysis between triolein (OOO) and glycerol	Acetonitrile; *t*-butanol; *n*-hexane; *n*-heptane; *n*-octane; THF; trichloromethane	Relatively hydrophobic solvent: high activity (except for acetonitrile)	[92]
Lipozyme TL IM	Esterification between monoolein and OA	*t*-Butanol; *t*-amyl alcohol; toluene; hexamethylene; *n*-hexane	Solvent with high log *P*: high activity and acyl migration, weak sn-1,3 selectivity decreased	[91]
Novozym 435	Ethanolysis of the fungal oil	Solventless; hexane; dichloromethane; acetone; *t*-butanol; ethanol	Solventless system: the highest acyl migration rate; polar solvent system: low acyl migration rate	[93]
Lipozyme RM IM	Acidolysis between tripalmitin and FFAs	Acetone; *t*-butanol; *n*-hexane	Polar solvent: low acyl migration	[26]
Lipozyme TL IM	Esterification between monoolein and OA	Acetone; *t*-butanol; trichloromethane; *n*-hexane	Solvent with high log *P*: high activity, weak sn-1,3 selectivity	[95]

It was reported that in many cases lipases expressed higher catalytic activity but lower sn-1,3 selectivity with increasing log *P* of the solvents [89–91]. Bi et al. [90] found that *Candida *sp. 99–125 lipase showed higher catalytic activity and lower sn-1,3 selectivity when increasing log *P* of the solvents. Duan et al. [89] observed the similar phenomenon when using Novozym 435 as the biocatalyst. The solvent effect on the regioselectivity was thought to be attributed to the variation of binding energy between lipase and different positions of hydroxyl groups [89–91]. On the contrary, whole-cell lipase from *Aspergillus niger* GZUF36 was found to exhibit stronger sn-1,3 selectivity in the solvent with higher log *P* value [92]. In some studies it was found that solvent can inhibit acyl migration, especially solvents with high log *P*, and adopting polar solvent as the reaction medium or adding a small amount of water was proposed for inhibiting the acyl migration [26, 36, 91]. While Wang et al. [93] revealed that the isomerization of 2-MAG was much faster in solvent-free system and *t-*butanol expressed obvious inhibition on the acyl migration.

Apart from solvent, water activity also plays a crucial role in lipase-mediated acylation reaction for the synthesis of HMFS. Different lipase had varied optimal water activity in terms of enzymatic activity and substrate specificity. Duan et al. [96] concluded that Novozym 435 exhibited strong activity and selectivity when the water activity was 0.53 during the esterification of oleic acid with glycerol. Wang et al. [95] found that Lipozyme TL IM showed the best performance at water activity near 0.33 in the esterification process of monoolein and oleic acid. Water activity may has complicated effect on lipase’s conformation flexibility and acyl migration, subsequently influencing the lipase’s activity and selectivity. Peng et al. [97] found that low water activity led to a high rate of acyl migration and the rate would decrease with the increase of water activity. Li et al. [98] reported that high water activity increased the charge dispersion of the transition state, which would increase the reaction activation energy, and ultimately led to the decrease of acyl migration rate. While Oh et al. [83] observed a significant increase in acyl migration rate when water activity increased from 0.22 to 0.65 in lipase-catalyzed acidolysis between olive oil and capric acid. Chen et al. [99] reported that during Lipozyme RM IM-catalyzed acidolysis the degree of acyl migration was the lowest with water activity of 0.55. The similar result was also reported by Xiao et al. [26], where the degree of acyl migration was found to be the lowest at water activity of 0.53, compared to 0.11 and 0.97. To provide a rational guidance for specified lipase-mediated HMFS production, more studies need to be investigated especially in terms of revealing the influence mechanism of water activity.

## Conclusions and foresight

As energy supplements, sources of essential fatty acids and nutritional supplements in infant formulas, HMFSs have been drawing increasing interest in recent years. Several HMFSs such as OPO, OPL, and HMFS enriched in long-chain polyunsaturated fatty acids, MLCT-type HMFS, and HMFS similar to HMF have been successfully prepared and some of them have even been commercialized through enzymatic one-step, two-step or multiple-step approaches. It is no doubt that lipase-catalyzed HMFS preparation has a great prospect especially for the production of HMFS with desired nutritional and functional properties. However, to promote the wide application of lipase-mediated HMFS production, there are some challenges need to be further tackled. One of the biggest challenges is to mimic the complete nutritional requirements contained in breast milk fat, since it has been reported that HMF contains more than two hundred kinds of fatty acids. Apart from the diversity of fatty acids, it is found that these fatty acids are attached to numerous complex lipids including triglycerides, glycerophospholipids, sphingolipids and sterol lipids. So far, most studies are just focused on the synthesis of triglycerides, such as OPO, OPL and MLCT-type HMFS. Few studies are carried out for the synthesis of other lipids, such as glycerophospholipids, sphingolipids and sterol lipids, which may play a unique and valuable function for infants’ growth. Regarding these complex lipids, their influence on infants’ development as well as the related enzyme-mediated production technology needs to be investigated further. Other challenges, such as exploring novel natural substrates, developing higher efficient enzyme catalyst and realizing continuous large-scale production should be taken into consideration.

## Data Availability

Not applicable.

## Abbreviations

HMFS: Human milk fat substitutes
HMF: Human milk fat
TAG: Triacylglycerol
PA: Palmitic acid
OA: Oleic acid
LA: Linoleic acid
LCFA: Long-chain fatty acid
MCFA: Medium-chain fatty acid
PUFA: Long-chain polyunsaturated fatty acid
MLCT: Medium- and long-chain triglyceride
PPP: Tripalmitin
FFA: Free fatty acids
OPO: Oleoyl-2-palmitoyl-3-oleoylglycerol
OPL: Oleoyl-2-palmitoyl-3-linoleoylglycerol
ALA: α-Linolenic acid
AA/ARA: Arachidonic acid
EPA: Eicosapentaenoic acid
DHA: Docosahexaenoic acid
OMO: 1,3-Oleic-2-medium chain
MAG: Monoglyceride
USU: Unsaturated–saturated-unsaturated
SFA: Saturated fatty acid
FAEE: Fatty acid ethyl ester
MUFA: Monounsaturated fatty acid
CRL: Lipases from candida rugosa
BCL: Lipase from burkholderia cepacian
PPL: Lipase from porcine pancreas
DAG: Diglycerid
NCM: Non-catalyzed mechanism
LCM: Lipase-catalyzed mechanism
THF: Tetrahydrofuran
OOO: Triolei
